# Dynamic Deposition of Histone Variant H3.3 Accompanies Developmental Remodeling of the *Arabidopsis* Transcriptome

**DOI:** 10.1371/journal.pgen.1002658

**Published:** 2012-05-03

**Authors:** Heike Wollmann, Sarah Holec, Keith Alden, Neil D. Clarke, Pierre-Étienne Jacques, Frédéric Berger

**Affiliations:** 1 Temasek Lifesciences Laboratory, National University of Singapore, Singapore, Singapore; 2 Genome Institute of Singapore, Singapore, Singapore; 3 Department of Biological Sciences, National University of Singapore, Singapore, Singapore; National Institute of Genetics, Japan

## Abstract

In animals, replication-coupled histone H3.1 can be distinguished from replication-independent histone H3.3. H3.3 variants are enriched at active genes and their promoters. Furthermore, H3.3 is specifically incorporated upon gene activation. Histone H3 variants evolved independently in plants and animals, and it is unclear whether different replication-independent H3.3 variants developed similar properties in both phyla. We studied *Arabidopsis* H3 variants in order to find core properties of this class of histones. Here we present genome-wide maps of H3.3 and H3.1 enrichment and the dynamic changes of their profiles upon cell division arrest. We find H3.3 enrichment to positively correlate with gene expression and to be biased towards the transcription termination site. In contrast with H3.1, heterochromatic regions are mostly depleted of H3.3. We report that, *in planta*, dynamic changes in H3.3 profiles are associated with the extensive remodeling of the transcriptome that occurs during cell differentiation. We propose that H3.3 dynamics are linked to transcription and are involved in resetting covalent histone marks at a genomic scale during plant development. Our study suggests that H3 variants properties likely result from functionally convergent evolution.

## Author Summary

Histone proteins are assembled into nucleosomes to build the skeleton of chromosomes. Beyond their role as DNA scaffold, histones participate in the regulation of gene activity. Studies in animals have shown that the deposition of two different histone H3 variants, H3.1 and H3.3, requires distinct pathways and results in distinct profiles throughout the genome. H3 variants evolved independently in plants and animals. Hence, H3 variants' properties shared by plants and animals would reflect core functions that have been selected during evolution. Our study indicates that these core properties include the high enrichment of H3.3 at active genes and a relative low deposition of H3.3 over regions deprived of genes or with inactive genes. In contrast with H3.1, H3.3 incorporation is dynamic and accompanies global changes of gene activity at major developmental transitions. We anticipate that the dynamic link between H3.3 variants and transcription enables remodeling of histone modifications that contribute to developmental transitions.

## Introduction

Histones are not static scaffolding proteins but dynamic actors involved in many aspects of chromatin related functions. They are targets of chromatin modifiers that deposit covalent modifications on histone tails and thereby influence chromatin properties and affect transcriptional and translational activities. Histones H3 can be subdivided into several classes. In addition to the centromeric variant CENH3 [1], the variants H3.1 and H3.3 are highly similar in their amino acid composition, yet they are incorporated into the chromatin through different pathways [2], [3]. H3.1 is predominantly expressed and therefore incorporated during DNA replication, while H3.3 is deposited throughout the cell cycle [4]–[8].

To date, many studies in animal species show that H3.3, in contrast to H3.1, is distinctly distributed along the genome. *Drosophila* H3.3 is enriched in euchromatic regions, at loci of active gene expression [3]. Induction of gene expression leads to H3.3 enrichment, a process that is linked to transcription [9]. H3.3 densities over genes correlate with those of RNA polymerase II (RNAPII) [10], [11]. Similarly, mammalian H3.3 is enriched at actively transcribed genes, correlating with the presence of RNAPII [12]–[14]. As observed in *Drosophila*, induction of gene expression leads to enrichment of H3.3 [15], suggesting that H3.3 deposition over active genes might be driven by nucleosome displacement in the course of transcription [10].

In mammals, H3.3 enrichment has also been detected over telomeres, repressed genes and pericentric heterochromatin, resulting from deposition by distinct chaperone complexes [13], [16]–[18]. Furthermore, H3.3 marks the boundaries of cis-regulatory elements and is enriched over promoters of actively transcribed genes in *Drosophila* and mammals [12], [19], [20], indicating that the H3.3 enrichment over active genes and their promoters is a common and conserved feature of H3.3 in animal species.

Based on DNA sequences and gene structure, it is clear that H3.1 and H3.3 have evolved separately in animals and plants [21]–[25]. Yet, in both groups four amino acid changes distinguish the two H3 classes. While three of these four changes are located at positions 31, 87 and 90 in animals and plants, the actual amino acid changes are different. Both animal and plant H3.1 genes do not contain introns, but plant H3.1 genes are not organized in clusters like animal H3.1 genes [26]. As a result, plant H3.1 (and H3.3) transcripts are polyadenylated [27].

In contrast to animals, the knowledge about plant histone H3 is limited. Although it is likely that plant H3.1 expression is coupled to the cell cycle as in animals [22], [28]–[31], it has not been demonstrated clearly that H3 variant incorporation to chromatin is cell cycle regulated. H3 dynamics have been associated with a potential reprogramming event in the zygote [31]. Although it was demonstrated that amino acid residues 87 and 90 are essential [32] the mechanisms of H3.3 incorporation remain unclear and its link with transcriptional activity has not been established in plants. Here, we present the first genome-wide map of *Arabidopsis* H3.3 and H3.1 enrichment in chromatin and clarify their specificities regarding genomic features and cell cycle regulation.

## Results/Discussion

To generate genome-wide maps of H3 variant localization, we performed Chromatin-Immunoprecipitation followed by deep sequencing (ChIP-Seq). We detected the localization of fusion proteins between a green fluorescent protein (GFP) tag and histone variants expressed under their endogenous promoter in transgenic plants. As a positive control, we used an H3 antibody recognizing the C-terminal part of H3, while an anti-IgG antibody was used as a negative control. The *Arabidopsis* genome contains three H3.3 genes encoding the same protein and we tagged *HTR5* (*HISTONE THREE RELATED 5*), the most highly expressed gene of the family [31]. Similarly, we tagged *HTR13*, one of the five genes encoding the unique H3.1 protein in *Arabidopsis* [31]. The protein fusions HTR5::GFP and HTR13::GFP will hereafter be referred to as H3.3 and H3.1, respectively. A previous study in mammals has efficiently used GFP-family tags (EYFP) to perform ChIP and detected genome wide H3.1 and H3.3 incorporation [13]. Although the EYFP tag is rather large, this previous study did not report significant differences between the localization of H3 variants fused to EYFP or to HA tags [13], prompting us to use GFP tags in our study. In order to investigate H3 variant deposition dynamics during development, we harvested two types of tissue. First we used a sample comprising the meristem with leaf primordia and young leaves, which are enriched in cells still undergoing cell division (hereafter referred to as “dividing tissue”). We compared our results to those obtained from mature leaves harboring mostly differentiated, non-dividing cells (hereafter referred to as “non-dividing tissue”). Two biological replicates were generated for each sample (Figure S1, Table S1).

### H3 Variants Mark Different Genomic Features

**Figure 1:**
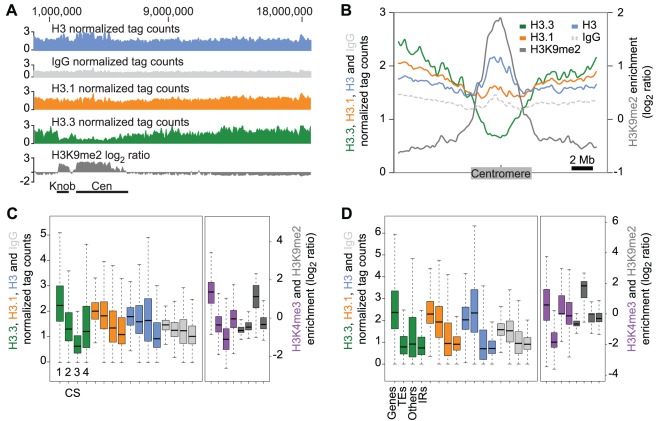
Genomic distribution of H3.3 and H3.1 enrichment over distinct chromatin states and genomic features. (A) Smoothed density of H3 (blue), IgG (light grey), H3.1 (orange), H3.3 (green) and H3K9me2 (dark grey), a heterochromatin mark, over the complete chromosome 4. The heterochromatic knob and centromeric regions are shown as black bars. Note the anti-correlation of H3.3 and H3K9me2 at the heterochromatic knob and over the centromere. (B) Average profile of H3.3 (green), H3.1 (orange), H3 (blue), IgG (light dashed grey) and H3K9me2 (dark grey) over 20 Mb genomic regions of the five chromosomes, centered on the middle of their centromeres. The anti-correlation between H3K9me2 and H3.3 enrichment is general over all centromeres. (C–D) Boxplot representations of the average enrichment of H3.3 (green), H3.1 (orange), H3 (blue), IgG (light grey), H3K4me3 (purple) and H3K9me2 (dark grey). (C) Distribution over chromatin states defined by [37]: 1545 CS1 regions mostly associated with H3K4me3, H3K9me3 and H3K36me3 active marks, 1046 CS2 regions associated mostly with H3K27me3 and H3K27me2 repressive marks, 637 CS3 regions associated mostly with H3K9me2, H4K20me1 and H3K27me1 constitutive heterochromatin marks, and 2413 CS4 regions with no prevalent marks. H3.3 is preferentially associated with CS1 and less with CS3 whereas H3.1 is more uniformly distributed. (D) Distribution over all genomic features annotated in TAIR9: 27337 protein-coding genes (Genes), 4838 transposable elements and pseudogenes (TEs), 1392 other annotations (Others), and 32910 inter-annotation regions >150 bp (IRs). H3.3 enrichment is much lower than H3.1 over TEs, and both H3 variants are more associated with genes than with inter-annotation regions.

We investigated the global distribution of H3.3 in comparison to H3.1 on major genomic features in dividing tissue (Figure 1). A browser view of the complete chromosome 4 showed that H3.3 signal decreased over the centromeric region in comparison to the chromosome arms (Figure 1A, green). Similarly, low H3.3 enrichment was observed on average around the centromeres of all five *Arabidopsis* nuclear chromosomes (Figure 1B, green). In contrast, H3.1 showed a more uniform signal along the genome (Figure 1A and 1B, orange). H3 levels were slightly increased over the centromeres (Figure 1A and 1B, blue), which was not surprising considering that nucleosome density has been reported to increase over pericentromeric regions [33]. Histone H3 lysine 9 dimethylation (H3K9me2) is a typical mark of constitutive heterochromatic regions found at the centromeres [34], [35]. We observed a clear anti-correlation between H3.3 and previously published H3K9me2 enrichment [36] over centromeres and at the heterochromatic knob (Figure 1A and 1B, grey).

We tested whether the anti-correlation between H3K9me2 and H3.3 was consistent at other smaller heterochromatic domains scattered along the chromosome arms. We used as a reference the four chromatin states (CS1 to CS4), defined in a previous study combining twelve different covalent histone marks [37]. We compared the distribution of H3.3 and H3.1 enrichment over these regions with the distribution of two histone modifications that mark active (H3K4me3) and inactive domains (H3K9me2) [37] (Figure 1C). CS3 is mostly enriched in H3K9me2, H4K20me1 and H3K27me1, and predominantly contains transposable element (TE) sequences [37]. CS3 thereby defines regions of constitutive heterochromatin, including the centromere and the heterochromatic knob. We observed that CS3 showed the lowest H3.3 levels among the four chromatin states. H3K4me3 levels are also low in CS3 regions [37] (Figure 1C).

CS1 and CS2 are predominantly associated with genes [37]. CS1 regions are mostly enriched in H3K4me3, H3K9me3 and H3K36me3 [37] (Figure 1C) and mark active genes, while CS2 domains are mostly enriched in marks associated with transcriptional repression (i.e. H3K27me3 and H3K27me2) [37]. H3.1 enrichment was quite similar over active (CS1) and repressed genes (CS2). Conversely, H3.3 appeared to be preferentially associated with CS1 rather than CS2 (Figure 1C). CS4, which defines regions without any prevalent histone mark [37], did not show preferential enrichment of H3.3 or H3.1, both profiles being similar to the H3 and IgG control profiles (Figure 1C). Overall, H3.1 was more evenly distributed than H3.3 over the different chromatin states (Figure 1C).

These results motivated the analysis of the H3 variant distribution over general genomic features including protein-coding genes, TEs and inter-annotation regions (IRs) (Figure 1D). The median values of H3.3 and H3.1 were similar over each feature, except over TEs where H3.3 enrichment was much lower than that of H3.1. This was consistent with poor H3.3 enrichment over TE-enriched CS3 (Figure 1C). Both H3 variants were associated similarly with IRs (Figure 1D). Notably, we obtained similar results for the H3.3 and H3.1 enrichment in dividing (Figure 1) and non-dividing (Figure S2) tissue. Moreover, there was no enrichment of either H3.3 or H3.1 at potential transcription factors binding sites (TFBS) from non-exonic regions (Figure S3).

In summary, the even distribution of H3.1 over all genomic features, similar to H3, suggests that H3.1 serves as a rather static chromatin backbone. In contrast, H3.3 appears to be more associated with active genes (CS1) than with repressed ones (CS2) and is depleted in regions of constitutive heterochromatin (CS3), including centromeres and TEs. H3.3 deposition at active genes appears to be conserved in yeast, *Drosophila* and mammals [10], [12], [13],[38] and our results suggest that plant H3.3 shares this common feature.

### Correlation of H3.3 Enrichment with Gene Expression

**Figure 2:**
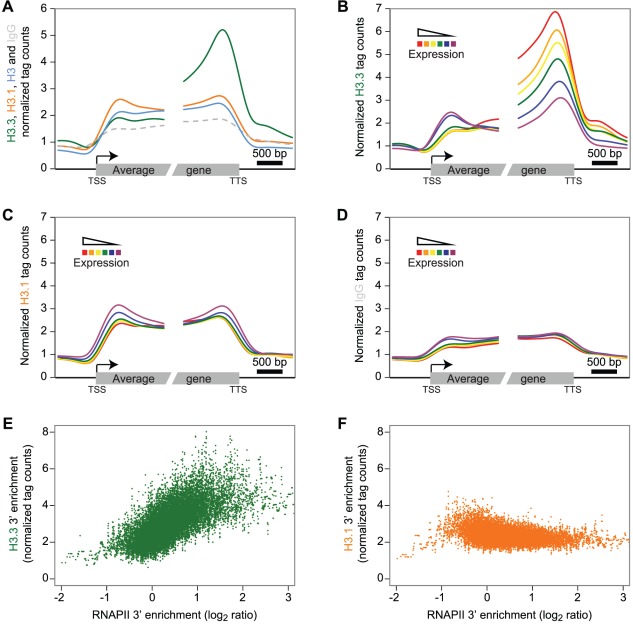
H3.3 enrichment profile over genes correlates with expression and is biased towards the 3′ end. (A) Average profile of H3.3 (green), H3.1 (orange), H3 (blue) and IgG (dashed grey) over gene bodies (all 14048 expressed protein-coding genes). Only the H3.3 profile peaks towards the 3′ end of the transcribed sequences. (B–D) Average profile of H3.3 (B), H3.1 (C) and IgG (D) enrichment over the protein-coding genes grouped according to their expression levels into six different subsets (from the red to the purple curves corresponding to FPKM >30, 20–30, 10–20, 5–10, 1–5, 0–1, and containing 3179, 1463, 2897, 2344, 2780 and 1263 genes, respectively). Note the strong correlation between levels of expression and H3.3 enrichment (B). By contrast, H3.1 enrichment does not appear to be linked with transcription (C). (E–F) Scatterplots of the H3.3 (E) and H3.1 (F) versus RNAPII 3′ enrichment calculated on the last 1 kb of the genes. A sliding window of 10 genes was applied on both H3 variants and RNAPII enrichment. Only H3.3 is showing a positive correlation with RNAPII.

We next investigated the enrichment profiles of the H3 variants over protein-coding genes and their flanking intergenic sequences in dividing tissue (Figure 2A–2D). To reveal a potential preference of H3.3 enrichment at either end of the gene body, we aligned the 5′ half of all genes at their transcription start site (TSS) and their 3′ half at their transcription termination site (TTS). H3.3 signal over genes showed a marked increase towards the 3′ end (Figure 2A, green). In contrast, H3.1 did not display preferential enrichment at either gene end (Figure 2A, orange), neither was a preferential enrichment observed for the H3 or IgG profiles (Figure 2A, blue and dashed gray, respectively). Also noteworthy, both H3 variants appear to distinctly mark the gene bodies compared to their 5′ and 3′ flanking regions.

According to our results on CS1 and CS2 (Figure 1C), H3.3 might be more enriched at actively transcribed genes. We tested this hypothesis by sequencing the transcripts (mRNA-Seq) from tissue corresponding to that used for ChIP-Seq analysis (Table S1). We grouped protein-coding genes into six subsets according to their expression levels and computed, for each expression group, the average profile of the H3 variant enrichment over genes. The level of H3.3 enrichment at the 3′ end of genes correlated positively with gene expression (Spearman rank correlation of 0.53 across all genes; enrichment calculated on the 3′ last 1 kb) (Figure 2B). At the 5′ end, there was no such positive correlation; if anything, we observed a slight negative correlation. In contrast to H3.3, H3.1 enrichment did not correlate with gene expression levels (Figure 2C), neither did we detect a correlation with the control profiles of IgG (Figure 2D) or H3 (Figure S4A). Similar results were observed in non-dividing tissue (Figure S5).

In agreement with our observations, plant H3.3 is associated with several histone marks, which are correlated with active gene expression [24], [39], [40]. In contrast to H3.3 however, profiles of euchromatic histone modifications do not appear to show preferential 3′ enrichment over genes (Figure S4B) [37], [41].

Animal H3.3 enrichment shows a positive correlation to gene expression [13], [14], [42] and plant and animal H3.3 appear to share this common feature. Several studies report animal H3.3 enrichment to correlate with that of RNA polymerase II (RNAPII) [10], [11], [14]. Therefore we analyzed *Arabidopsis* RNAPII enrichment over genes using data published previously [33] and indeed found a 3′ preference (Figure S4C). Moreover, the enrichment of H3.3 and RNAPII calculated on the 3′ last 1 kb was positively correlated (Figure 2E, Spearman rank correlation of 0.33 across all genes, p-value<1e-275), even if the RNAPII was not profiled in the same condition. This was obviously not the case for H3.1, H3 or IgG (Figure 2F, Figure S4D–S4E). We noticed that the enrichment of H3.3 at the 3′ end also positively correlated with gene length, whereas H3.1 and RNAPII did not (Figure S6A–S6E).

In summary, plant H3.3 enrichment positively correlates with gene expression and gene length and appears to gradually increase towards the distal gene end, reaching a maximum immediately upstream of the TTS. This profile appears to be similar to that reported for *C. elegans* H3.3 [43]. Preferential enrichment of H3.3 towards the gene end has also been reported in mouse cells, where activation of interferon-stimulated genes leads to H3.3 incorporation preferentially at the distal coding region [15]. Similarly, in human cells H3.3 abundance appeared to show a gradual increase towards the TTS [42]. A recent study that compares H3.3 patterns over genes in mouse and human cells reports H3.3 enrichment to be highest after the TTS, a profile that correlates with that of RNAPII [14]. In plants, we find that the H3.3 enrichment at the 3′ end broadly correlates with the RNAPII profile. Hence both in plants and animals H3.3 deposition appears to be linked to or to enable co-transcriptional processes but whether this reflects similar mechanisms remains to be investigated.

While plant and animal H3.3 obviously share similar features, we observed important differences as well. H3.3 enrichment in *Drosophila* and mammals is not limited to the coding regions but is also high on cis-regulatory elements, repressed genes and telomeres [11]–[13], [19],[20], which appears not to be true for plant H3.3. This might be explained by a regulatory function of H3.3 in animals that has not evolved similarly in plants. The absence of enrichment of H3.3 in non-coding regions might also reflect that the *Arabidopsis* genome lacks long distance acting enhancers, which are common in mammals and *Drosophila*.

### Dynamic H3.3 Replacement during Developmental Transition

We investigated the dynamics of H3.3 enrichment during the major developmental transition in vegetative plant life that leads to leaf formation. Leaf development is initiated from primordia that continuously arise from the shoot apical meristem (SAM). The SAM and the primordia comprise dividing cells [44]. Once a primordium enlarges through cell division, leaf patterning takes place while cells still divide. Subsequent cell differentiation coincides largely with the arrest of cell division. Thus, we compared H3 variant enrichment in meristem and leaf primordia (dividing tissue) and mature leaves (non-dividing tissue). Using data from cyclebase.org [45] and the transcriptomes obtained from each sample, we verified that dividing tissues expressed cell cycle regulated genes, including the five genes encoding H3.1 variants at levels higher than non-dividing tissues (Table 1 and Table S2). In animals, incorporation of H3.1 and H3.3 into chromatin depends on distinct assembly factors. While ASF1A and ASF1B are apparently required for deposition of both H3 types, the CAF-1 complex participates in H3.1 incorporation, while H3.3 incorporation depends on HIRA and DAXX [8], [13], [18], [46]. Except for DAXX, homologues of the H3 chaperones have been identified in the *Arabidopsis* genome (Table 1). Amongst these homologues, only the expression of the H3.1-specific CAF1 homolog *FAS2* was strongly dependent on the cell cycle (Table 1). Together, the expression profiles of the H3 variants and their chaperones suggest that in *Arabidopsis*, as is the case in animals, H3.1 incorporation occurs primarily in dividing cells while H3.3 incorporation is largely independent of the cell cycle.

**Table 1:** Expression of histone H3 and potential histone H3 chaperone genes in dividing and non-dividing tissue.

Category	Gene	Name	CycleBase Rank	Dividing (FPKM)	Non-dividing (FPKM)	Ratio Div/Non
**H3.3**	AT4G40030	HTR4	11,291	464.6	286.5	
	AT4G40040	HTR5	4,674	644.0	351.4	
	AT5G10980	HTR8	7,701	154.6	125.7	
	**total**			**1263.1**	**763.6**	**1.65**
**H3.1**	AT1G09200	HTR2	171	53.4	16.1	
	AT3G27360	HTR3	78	19.5	3.6	
	AT5G10390	HTR13	44	16.4	4.4	
	AT5G10400	HTR9	661	66.5	12.7	
	AT5G65360	HTR1	262	58.6	22.5	
	**total**			**214.4**	**59.4**	**3.61**
**cenH3**	AT1G01370	HTR12	1,848	3.9	0.7	**5.59**
**H3 chaperones**	AT3G44530	HIRA	13,620	5.7	3.8	
	AT1G08600	ATRX	14,566	9.4	6.5	
	AT1G66740	ASF1A	10,888	15.9	13.1	
	AT5G38110	ASF1B	15,230	14.0	3.9	
	AT5G58230	MSI1	6,931	17.3	12.1	
	AT1G65470	FAS1	340	3.2	2.0	
	AT5G64630	FAS2	2,243	5.2	0.7	
	**total**			**70.7**	**42.1**	**1.68**

CycleBase ranks were extracted from www.cyclebase.org, with ranks from 1 to 20,945 reflecting the magnitude of cell-cycle dependent regulation. *H3.3* genes for which we did not detect expression are not presented here. FPKM values are the average of the two mRNA-Seq replicates per tissue, except for HTR5 and HTR13 where only the values from the non-tagged library were reported.

**Figure 3:**
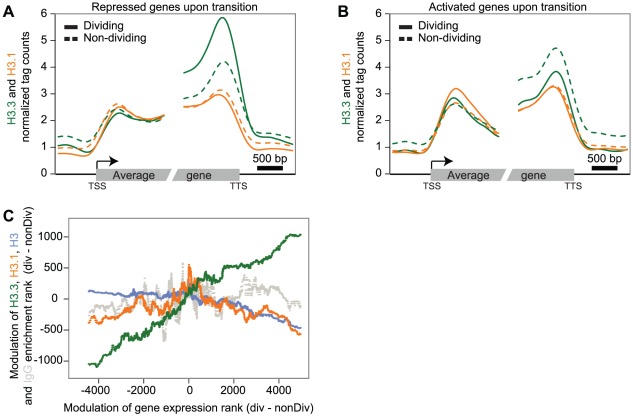
Dynamics of H3.3 and H3.1 enrichment during development. (A–B) Average profile of H3.3 (green) and H3.1 (orange) enrichment over genes that are five fold up-regulated in dividing tissue (plain line) compared to non-dividing tissue (dashed line) (194 genes) (A) and vice versa (88 genes) (B). The average H3.3 enrichment varies during development and follows expression changes (A–B). In contrast, the average H3.1 enrichment does not vary significantly during the developmental transition (A–B). (C) Scatterplots of the modulation of H3.3 (green), H3.1 (orange), H3 (blue) and IgG (dashed grey) enrichment rank versus gene expression rank in dividing compared to non-dividing tissue. A sliding window of 500 genes was applied on both the gene expression difference and the differential enrichment. Only the modulation of H3.3 shows a positive correlation with changes of levels of expression.

To investigate H3.3 and H3.1 dynamics during the developmental transition from primordia to differentiated leaves, we selected two subsets of genes, according to their higher expression levels (at least five-fold) in either dividing or non-dividing tissue. Having gene sets that were preferentially expressed in either of the two tissue types, we could examine the changes in H3 variant levels that accompanied repression (Figure 3A) and induction (Figure 3B) of transcription during the developmental transition from dividing tissues (plain lines) to non-dividing tissues (dashed lines). Transcriptional repression was accompanied by a strong decrease of H3.3 levels at the 3′ end (Figure 3A). Conversely, activation of gene transcription at the developmental transition was reflected in an increase of H3.3 signal at the 3′ end (Figure 3B). H3.1 levels on the other hand were not affected at genes undergoing repression (Figure 3A) or activation (Figure 3B). Similarly, different groups of genes (cell cycle regulated genes, genes expressed in only one tissue, and control genes with similar expression) also supported that H3.3 enrichment changed dynamically according to the expression modulation (Figure S7). Moreover, when considering all the genes, we observed a positive correlation between expression change and the change in H3.3 enrichment that is modest, but highly significant (Spearman rank correlation of 0.28, p-value<1e-275) (Figure 3C). This was not the case for H3.1, H3 and IgG (Figure 3C).

We conclude that the repression of gene expression during leaf differentiation is linked with a decrease in the H3.3 level, but not H3.1 level, suggesting that H3.3 may contribute to developmental transitions. Differentiation also requires the induction of gene expression, which correlates with gain of H3.3 enrichment at the 3′ end of some genes. H3.1 enrichment on the other hand, is not significantly affected by developmental transitions and appears to be a relatively stable chromatin component. This property would support a role of H3.1 in propagation of epigenetic patterns of histone modification through division, in agreement with the preference of H3.1 over H3.3 enrichment at heterochromatic regions, which need to be maintained in a transcriptional silent state.

### Conclusions

There are remarkable similarities between H3 variants in animals and plants, yet phylogenetic analyses indicate that amongst metazoa and plantae, H3.1 and H3.3 variants evolved independently. However, both share similar features such as specific amino acid changes at positions 31 and 87 and the absence of introns in H3.1 [21], [23], [25]. This suggested that H3 variants in plants and animals are analogous and result from convergent evolution of similar H3 properties. In both, plants and animals H3.1 but not H3.3 expression is linked to the cell cycle [21], [47]. Furthermore, our results indicate that in *Arabidopsis* H3.3 is dynamically deposited over gene bodies and its enrichment is linked to gene expression. Thus, the incorporation specificities observed for H3.3 and H3.1 are largely similar between animals and plants, suggesting a functional convergence during evolution of H3 variants in eukaryotes. Whether this convergence was driven by the conservation of the distinct mechanisms that incorporate H3.1 and H3.3 remains to be established since we currently lack biochemical characterization of histone H3 chaperones in plants.

Our study outlines a specific enrichment of H3.3 culminating towards the 3′ end of genes, a phenomenon that might be linked with gene length. Noteworthy, active marks such as H3K4me3 and H3K4me36 are enriched towards the 5′ part of genes, in contrast to H3.3. Although we observe a correlation with RNAPII, the origin and significance of H3.3 enrichment at the 3′ end of genes in *Arabidopsis* remains unclear.

Our study addresses genome-wide dynamics of H3.3 and H3.1 enrichment during differentiation *in planta*. We find that gene expression changes during differentiation are reflected in H3.3 enrichment. This dynamic replacement of H3.3 potentially allows covalent histone marks present in the chromatin of dividing cells to be remodeled in order to allow repression or expression of a new repertoire of genes that participate to the differentiation program. Hence, H3 variant replacement might serve as a mechanism that enables reprogramming at developmental transitions by globally facilitating dynamics of covalent marks.

## Materials and Methods

### Plant Material and Growth Conditions

We used homozygous transgenic plants constructed by Mathieu Ingouff [31]. Columbia plants were agro-transfected with pHTR13:HTR13::GFP (At5g10390) for the tagged H3.1 line and with pHTR5:HTR5::GFP (At4g40040) for the tagged H3.3 line, using the destination vector pMDC107 [48], as described in [31]. Plants were grown in short day conditions (8 h light–16 h dark, 20 to 22°C) for 4 weeks after stratification at 4°C and in dark for 5 days. For harvesting the tissues used for ChIP and RNA sequencing, we dissected the plants with scalpels under a binocular scope. For the “dividing tissue” samples, we harvested the meristem and younger leaves, for the “non-dividing tissue” samples, we harvested the oldest 4 to 6 leaves.

### Chromatin Immunoprecipitation (ChIP)

Nuclei enrichment was performed as previously described [49] with modifications. Tissues grinded in liquid nitrogen were fixed in 1% formaldehyde for 10 minutes, and the reaction was stopped by adding 0.125 M glycine. Nuclei were extracted by filtration through Miracloth and iterated washes and centrifugations at 2,000×g. Chromatin Immunoprecipitation was done as previously described [50] with modifications. After lysis in SDS buffer, DNA was sonicated for 8 cycles of 0.5 minute on and 1 minute off with an UCD-200TM-EX Bioruptor (Diagenode) on medium power, at 0 to 4°C. Sonicated chromatin was incubated overnight with either GFP antibodies (A11122, Invitrogen), H3 antibodies (07-690, Millipore), or IgG antibodies (ab46540-1, Abcam). After pre-clearing, magnetic protein A-beads (Dynabeads protein A, Invitrogen) were incubated with the antibodies-chromatin mix for 3 hours. After precipitation of the beads on a magnetic rack (MagnaRack, Invitrogen) and washes with increasing stringency, DNA was eluted at 65°C and reverse cross-linked with proteinase K (Fermentas). Immunoprecipitated DNA was treated with RNase A (Fermentas) and purified with the QIAquick purification kit (Qiagen). For the first biological replicate (Table S1), DNA was reverse cross-linked with Chelex resin (BioRad) 10 minutes at 95°C, and the antibody used to immunoprecipitated H3 was ab1791 (Abcam).

### ChIP and RNA Sequencing

mRNA-Seq and ChIP-Seq libraries of template molecules suitable for high-throughput sequencing were constructed according to the guide lines described in the Illumina website (http://www.illumina.com/applications/sequencing/.ilmn). For mRNA-Seq libraries, briefly, 10 µg of total RNA was purified to yield poly-A containing mRNA molecules using poly-T oligo-attached magnetic beads. Following purification, the mRNA was fragmented into small pieces using divalent cations under elevated temperature. Then the cleaved RNA fragments were copied into first strand cDNA using Superscript II Reverse Transcriptase (Invitrogen) and random primers. This was then followed by second strand cDNA synthesis using DNA Polymerase I and RNaseH. These cDNA fragments then underwent an end repair process, the addition of a single ‘A’ base, and then ligation of adapters specific for sequencing flow cell. These products were then purified by gel excision and enriched by PCR with Phusion polymerase (Fermentas) to create the final cDNA library. This library was validated by loading 1 µl of the re-suspended constructs onto an Agilent Technologies 2100 Bioanalyzer DNA-1000 microfluidic chip. The final products showed a distinct band at 200–300 bp and were subsequently sequenced on an Illumina Genome Analyser IIx.

A similar process was used for ChIP-Seq libraries generation. 30 µl of chromatin-immunoprecipitated DNA was subjected to the following process: end repair by the addition of a single ‘A’ base, and then ligation of adapters specific for sequencing flow cell. These products were then purified and size-selected on gel to have fragments from 200 to 300 bp and enriched by 20 cycles PCR to create the final cDNA library (22 cycles for replicate 1). This library was validated by loading 1 µl of the re-suspended constructs onto an Agilent Technologies 2100 Bioanalyzer DNA-1000 microfluidic chip. The final products showed a distinct band at 200–300 bp and were subsequently sequenced on an Illumina Genome Analyser IIx.

### Reads Mapping

The ChIP-Seq reads were mapped onto the *Arabidopsis* genome (TAIR9) using CASAVA v1.7. The number of mapped reads varied from 228 k to 11.9 M for the first biological replicates, and from 15.1 M to 20.7 M for the second (Table S1).

These files were then converted to 10 bp density WIG files using MACS v1.4.0 [51]. In order to be directly comparable, each WIG file was next normalized using the total number of mapped reads. We performed visual inspection of the data using a local installation of the UCSC Genome Browser [52] (http://genome.gis.a-star.edu.sg/).

The mRNA-Seq reads were mapped onto TAIR9 using recommended settings of TopHat v1.2.0 [53], Bowtie v0.12.7 [54] and Samtools v0.1.13 [55]. The number of mapped reads varied from 34.6 M to 39.4 M (Table S1). These files were then analyzed with Cufflinks v0.9.3 [56] using recommended settings to get a FPKM (Fragment Per Kilobase per Million mapped reads) value for each annotation (Table S3).

The quality of the mapped reads was assessed using FastQC v.0.9.0 (http://www.bioinformatics.bbsrc.ac.uk/projects/fastqc/).

The raw reads and processed files from both ChIP-Seq and mRNA-Seq experiments have been deposited in the NCBI Gene Expression Omnibus (GEO) (http://www.ncbi.nlm.nih.gov/geo/) and are accessible through GEO series accession number GSE36631.

### Average Profiles

In order to generate the average profile over centromeres (Figure 1B and Figure S2B), the centers of the 5 centromeric regions (coordinates from [57]) were aligned and the average signal calculated into 200 kb windows over 20 Mb. An average sliding window of 1 Mb was next applied to the result. The average profiles over genes (Figure 2, Figure 3; Figures S4, S5, S6, S7) were similarly generated on the 5′ and 3′ transcriptional boundaries into 50 bp windows for each half-gene and adjacent inter-annotation regions. An average sliding window of 5 kb was next applied to the result. All these profiles were generated using a tool that will be available soon (Jacques et al., In preparation).

Note that we excluded from these analyses all mitochondrial and chloroplast genes as well as nuclear genes that overlap other annotations. We also excluded genes whose transcripts are shorter than 1 kb, except for the analysis shown in Figure S6 from which this exclusion criterion was derived (Table S3). Note that the total number of genes is not consistent because in Figure 2B, all genes expressed (FPKM>0) in at least one of the four mRNA-Seq library were used (for a total of 14048 after applying the other filters), while in the other cases we decided to be conservative and discarded ∼10% genes (3897 from 33476) with unexpected expression variation between replicates from the same tissue (variation being defined as either i) an absolute difference of FPKM higher than 1.0 (ex: FPKM of 3 in one replicate and 4.5 in the other; 1.5>1), ii) the proportion of this absolute FPKM difference was more than a third of the minimal FPKM (ex: FPKMs of 1 and 1.5; 0.5/1>0.33), iii) FPKM null in only one replicate).

### Boxplot Distributions and Spearman Correlations

The boxplot distributions were generated using the boxplot function of the graphics package v2.11.1 in R, on the average signal over each feature listed. The chromatin states (CS) regions used in Figure 1C and Figure S2C were derived from [37]. Adjacent regions sharing the same status were merged. The genomic features used in Figure 1D and Figure S2D were extracted from the TAIR9 annotation file. The p-values (two-sided) were calculated using the t.test function of the package stats in R. The Spearman correlations were computed on FPKM expression values vs H3.3 enrichment of the last kb of genes. Average rank was used in cases of tied values.

For the scatterplots of Figure 2E–2F and Figure S4D–S4E, a sliding window of 10 genes was applied on both H3 variants and RNAPII enrichment. For Figure 3C a sliding window of 500 genes was applied on both the gene expression difference (div – nonDiv) and the differential enrichment difference (div – nonDiv) after ordering the data on the RNAPII or expression data respectively.

All the data used in the boxplots and scatterplots are available in Table S4.

### Enhancers Analysis

Predicted Transcription Factor Binding Sites (TFBS) data from AthaMap database [58] containing ∼10 millions unique sites from 124 different matrices was converted to TAIR9 coordinates using the script “update_coordinates.pl” from “ftp://ftp.arabidopsis.org/home/tair/Software/UpdateCoord/”. Based on the authors suggestion, the number of sites per matrix was limited to 200,000 following the “restriction” procedure (http://www.athamap.de/restriction_scores.php), giving ∼4.7 millions sites. The 2,390,614 non-exonic sites were then used to generate an average profile as described above (50 bp windows over 2 kb, sliding window of 200 bp) and presented in Figure S3.

## Supporting Information

Figure S1 Genome browser snapshot of 31 kb on chromosome 3. Profiles of H3 (blue), H3.1 (orange), H3.3 (green) and IgG (grey) obtained from two independent biological replicates in the two tissue types. The region includes several protein-coding genes (black on positive strand, purple on negative strand) having the following average FPKM values per condition (div/nonDiv): AT3G14820 (0/0), AT3G14830 (18.6/19.7), AT3G14840 (19.8/38.2), AT3G14850 (1.7/1.1), AT3G14855 (0/0), AT3G14860 (12.0/13.9), AT3G14870 (1.4/3.7). Vertical dashed grey lines correspond to the TTS of the genes.
(EPS)

Figure S2 Genomic distribution of H3.3 and H3.1 enrichment in non-dividing tissue. (A–D) Replicate of Figure 1 showing non-dividing rather than dividing tissue. The gene annotation track (A, black points) is showing a similar overall profile than H3.3.
(EPS)

Figure S3 Genomic distribution of H3.3 and H3.1 enrichment over Transcription Factor Binding Sites (TFBS). Profiles of H3.3 (green), H3.1 (orange), H3 (blue) and IgG (dashed grey) over non-exonic TFBS extracted from AthaMap [58].
(EPS)

Figure S4 Enrichment profiles of marks and RNAPII over genes. (A) Average profile of H3 enrichment in dividing cells over the protein-coding genes grouped according to their expression levels into six different subsets (see Figure 2B–2D). (B) Average profile of H3K36me3 (red), H3K4me3 (purple), H3K9me2 (dark grey) and H3 (blue) enrichment over gene bodies (all 14048 expressed protein-coding genes) presented by Roudier and colleagues [37]. (C) Average profile of RNAPII enrichment [33] over the protein-coding genes. (D–E) Scatterplots of H3 (D) and IgG (E) versus RNAPII 3′ enrichment calculated on the last 1 kb of the genes. A sliding window of 10 genes was applied on both H3 variants and RNAPII enrichment.
(EPS)

Figure S5 H3.3 and H3.1 enrichment over genes in non-dividing tissue. (A) Average profile of H3.3 (green), H3.1 (orange), H3 (blue) and IgG (dashed grey) enrichment over gene bodies (all 14048 expressed protein-coding genes). (B–E) Average profile of H3.3 (B), H3.1 (C), H3 (D) and IgG (E) enrichment over the protein-coding genes grouped according to their expression levels into six different subsets (from the red to the purple curves corresponding to FPKM >30, 20–30, 10–20, 5–10, 1–5, 0–1, containing 3179, 1463, 2897, 2344, 2780 and 1263 genes, respectively).
(EPS)

Figure S6 Correlation of H3.1 and H3.3 enrichment with gene length. Average profile of H3.3 (A), H3.1 (B), H3 (C), IgG (D) from dividing tissue over the protein-coding genes grouped according to their length into five different subsets (from the red to the purple curves corresponding to >4 kb, 3 kb–4 kb, 2 kb–3 kb, 1 kb–2 kb, <1 kb, containing 2724, 3091, 6877, 8972 and 5463 genes, respectively). Non-dividing profiles (not shown) are almost identical.
(EPS)

Figure S7 Dynamics of H3.3 and H3.1 enrichment during development over different sets of genes. Average profile of H3.3 (green) and H3.1 (orange) enrichment in dividing tissue (plain line) compared to non-dividing tissue (dashed line) over genes that are cell cycle regulated according to CycleBase (A), genes that are ON (FPKM>3) in dividing tissue and OFF (FPKM<1) in non-dividing tissue (B) and vice-versa (C) and genes that present similar expression in both tissue types and are either highly expressed (D), or have low expression levels (E). (F–G) Snapshots of the UCSC genome browser showing H3 (blue), H3.1 (orange), H3.3 (green), IgG (grey) and mRNA-Seq (black) over representative protein-coding genes (black on positive strand, purple on negative strand) more expressed in dividing tissue than non-dividing tissue (F) or the opposite (G). The corresponding FPKM values per condition (div/nonDiv) are: AT3G14600 (126.9/422.4), AT3G14610 (1.3/2.4), AT3G14620 (8.7/3.3), AT1G20440 (1768.8/134.5), AT1G20450 (503.1/94.9). Horizontal dashed grey lines correspond to half the scale of each graph.
(EPS)

Table S1 Overview of sequencing libraries analyzed.
(DOC)

Table S2 Expression of the top-most cell-cycle dependent and independent genes in dividing and non-dividing tissue. CycleBase ranks were extracted from www.cyclebase.org with ranks from 1 to 20,945 reflecting the magnitude of cell-cycle dependent regulation. FPKM values are mean values obtained from the HTR5::GFP and HTR13::GFP mRNA-Seq libraries.
(DOC)

Table S3 Tab-delimited file containing FPKM values for each TAIR9 annotation. The last column contains the information whether the gene was kept or not for the downstream analyses based on expression variation between replicates from the same tissue (see Materials and Methods).
(GZ) Click here for additional data file.

Table S4 Tab-delimited file containing the average signal of all datasets used in the boxplots and scatterplots over each annotation (total and last kb), inter-annotation and CS segment.
(GZ) Click here for additional data file.

## References

[1] Dalal Y, Bui M (2010). Down the rabbit hole of centromere assembly and dynamics.. Curr Opin Cell Biol.

[2] Tagami H, Ray-gallet D, Almouzni G, Nakatani Y (2004). Histone H3.1 and H3.3 Complexes Mediate Nucleosome Assembly Pathways Dependent or Independent of DNA Synthesis.. Cell.

[3] Ahmad K, Henikoff S (2002). The histone variant H3.3 marks active chromatin by replication-independent nucleosome assembly.. Mol Cell.

[4] Ahmad K, Henikoff S (2002). Histone H3 variants specify modes of chromatin assembly.. Proc Natl Acad Sci U S A.

[5] Banaszynski LA, Allis CD, Lewis PW (2010). Histone variants in metazoan development.. Dev Cell.

[6] Henikoff S, McKittrick E, Ahmad K (2004). Epigenetics, histone H3 variants, and the inheritance of chromatin states.. Cold Spring Harb Symp Quant Biol.

[7] Talbert PB, Henikoff S (2010). Histone variants–ancient wrap artists of the epigenome.. Nat Rev Mol Cell Biol.

[8] Corpet A, Almouzni G (2009). Making copies of chromatin: the challenge of nucleosomal organization and epigenetic information.. Trends Cell Biol.

[9] Schwartz BE, Ahmad K (2005). Transcriptional activation triggers deposition and removal of the histone variant H3.3.. Genes Dev.

[10] Wirbelauer C, Bell O, Schübeler D (2005). Variant histone H3.3 is deposited at sites of nucleosomal displacement throughout transcribed genes while active histone modifications show a promoter-proximal bias.. Genes Dev.

[11] Mito Y, Henikoff JG, Henikoff S (2005). Genome-scale profiling of histone H3.3 replacement patterns.. Nat Genet.

[12] Daury L, Chailleux C, Bonvallet J, Trouche D (2006). Histone H3.3 deposition at E2F-regulated genes is linked to transcription.. EMBO reports.

[13] Goldberg AD, Banaszynski LA, Noh K-M, Lewis PW, Elsaesser SJ (2010). Distinct Factors Control Histone Variant H3.3 Localization at Specific Genomic Regions.. Cell.

[14] Ray-Gallet D, Woolfe A, Vassias I, Pellentz C, Lacoste N (2011). Dynamics of histone h3 deposition in vivo reveal a nucleosome gap-filling mechanism for h3.3 to maintain chromatin integrity.. Mol Cell.

[15] Tamura T, Smith M, Kanno T, Dasenbrock H, Nishiyama A (2009). Inducible deposition of the histone variant H3.3 in interferon-stimulated genes.. The Journal of biological chemistry.

[16] Drané P, Ouararhni K, Depaux A, Shuaib M, Hamiche A (2010). The death-associated protein DAXX is a novel histone chaperone involved in the replication-independent deposition of H3.3.. Genes Dev.

[17] Wong LH, McGhie JD, Sim M, Anderson MA, Ahn S (2010). ATRX interacts with H3.3 in maintaining telomere structural integrity in pluripotent embryonic stem cells.. Genome Res.

[18] Lewis PW, Elsaesser SJ, Noh KM, Stadler SC, Allis CD (2010). Daxx is an H3.3-specific histone chaperone and cooperates with ATRX in replication-independent chromatin assembly at telomeres.. Proc Natl Acad Sci U S A.

[19] Mito Y, Henikoff JG, Henikoff S (2007). Histone replacement marks the boundaries of cis-regulatory domains.. Science.

[20] Chow C-M, Georgiou A, Szutorisz H, Maia e Silva A, Pombo A (2005). Variant histone H3.3 marks promoters of transcriptionally active genes during mammalian cell division.. EMBO reports.

[21] Waterborg JH (2011). Evolution of histone H3: emergence of variants and conservation of post-translational modification sites.. Biochem Cell Biol.

[22] Waterborg JH, Robertson AJ (1996). Common features of analogous replacement histone H3 genes in animals and plants.. J Mol Evol.

[23] Robertson AJ, Kapros T, Dudits D, Waterborg JH (1996). Identification of three highly expressed replacement histone H3 genes of alfalfa.. DNA Seq.

[24] Waterborg JH (1991). Multiplicity of histone h3 variants in wheat, barley, rice, and maize.. Plant Physiol.

[25] Malik HS, Henikoff S (2003). Phylogenomics of the nucleosome.. Nat Struct Biol.

[26] Ingouff M, Berger F (2009). Histone3 variants in plants.. Chromosoma.

[27] Wu SC, Gyorgyey J, Dudits D (1989). Polyadenylated H3 histone transcripts and H3 histone variants in alfalfa.. Nucleic Acids Res.

[28] Chaubet N, Clement B, Gigot C (1992). Genes encoding a histone H3.3-like variant in Arabidopsis contain intervening sequences.. J Mol Biol.

[29] Lepetit M, Ehling M, Chaubet N, Gigot C (1992). A plant histone gene promoter can direct both replication-dependent and -independent gene expression in transgenic plants.. Mol Gen Genet.

[30] Okada T, Endo M, Singh MB, Bhalla PL (2005). Analysis of the histone H3 gene family in Arabidopsis and identification of the male-gamete-specific variant AtMGH3.. Plant J.

[31] Ingouff M, Rademacher S, Holec S, Soljic L, Xin N (2010). Zygotic resetting of the HISTONE 3 variant repertoire participates in epigenetic reprogramming in Arabidopsis.. Curr Biol.

[32] Shi L, Wang J, Hong F, Spector DL, Fang Y (2011). Four amino acids guide the assembly or disassembly of Arabidopsis histone H3.3-containing nucleosomes.. Proc Natl Acad Sci USA.

[33] Chodavarapu RK, Feng S, Bernatavichute YV, Chen PY, Stroud H (2010). Relationship between nucleosome positioning and DNA methylation.. Nature.

[34] Johnson L, Cao X, Jacobsen S (2002). Interplay between two epigenetic marks. DNA methylation and histone H3 lysine 9 methylation.. Curr Biol.

[35] Soppe WJ, Jasencakova Z, Houben A, Kakutani T, Meister A (2002). DNA methylation controls histone H3 lysine 9 methylation and heterochromatin assembly in Arabidopsis.. EMBO J.

[36] Turck F, Roudier F, Farrona S, Martin-Magniette ML, Guillaume E (2007). Arabidopsis TFL2/LHP1 specifically associates with genes marked by trimethylation of histone H3 lysine 27.. PLoS Genet.

[37] Roudier F, Ahmed I, Berard C, Sarazin A, Mary-Huard T (2011). Integrative epigenomic mapping defines four main chromatin states in Arabidopsis.. EMBO J.

[38] Choi ES, Shin JA, Kim HS, Jang YK (2005). Dynamic regulation of replication independent deposition of histone H3 in fission yeast.. Nucleic acids research.

[39] Johnson L, Mollah S, Garcia BA, Muratore TL, Shabanowitz J (2004). Mass spectrometry analysis of Arabidopsis histone H3 reveals distinct combinations of post-translational modifications.. Nucleic Acids Res.

[40] Waterborg JH (1990). Sequence Analysis of Acetylation and Methylation in Two Histone H3 Variants of Alfalfa *.. The Journal of biological chemistry.

[41] Zhang X, Bernatavichute YV, Cokus S, Pellegrini M, Jacobsen SE (2009). Genome-wide analysis of mono-, di- and trimethylation of histone H3 lysine 4 in Arabidopsis thaliana.. Genome Biol.

[42] Jin C, Zang C, Wei G, Cui K, Peng W (2009). H3.3/H2A.Z double variant-containing nucleosomes mark ‘nucleosome-free regions’ of active promoters and other regulatory regions.. Nature genetics.

[43] Ooi SL, Henikoff JG, Henikoff S (2010). A native chromatin purification system for epigenomic profiling in Caenorhabditis elegans.. Nucleic Acids Res.

[44] Donnelly PM, Bonetta D, Tsukaya H, Dengler RE, Dengler NG (1999). Cell cycling and cell enlargement in developing leaves of Arabidopsis.. Dev Biol.

[45] Gauthier NP, Jensen LJ, Wernersson R, Brunak S, Jensen TS (2010). Cyclebase.org: version 2.0, an updated comprehensive, multi-species repository of cell cycle experiments and derived analysis results.. Nucleic Acids Res.

[46] Elsaesser SJ, Goldberg AD, Allis CD (2010). New functions for an old variant: no substitute for histone H3.3.. Current opinion in genetics & development.

[47] Okada T, Singh MB, Bhalla PL (2006). Histone H3 variants in male gametic cells of lily and H3 methylation in mature pollen.. Plant Mol Biol.

[48] Curtis MD, Grossniklaus U (2003). A gateway cloning vector set for high-throughput functional analysis of genes in planta.. Plant Physiol.

[49] Ito T, Takahashi N, Shimura Y, Okada K (1997). A serine/threonine protein kinase gene isolated by an in vivo binding procedure using the Arabidopsis floral homeotic gene product, AGAMOUS.. Plant Cell Physiol.

[50] Gendrel AV, Lippman Z, Martienssen R, Colot V (2005). Profiling histone modification patterns in plants using genomic tiling microarrays.. Nat Methods.

[51] Zhang Y, Liu T, Meyer CA, Eeckhoute J, Johnson DS (2008). Model-based analysis of ChIP-Seq (MACS).. Genome Biol.

[52] Fujita PA, Rhead B, Zweig AS, Hinrichs AS, Karolchik D (2011). The UCSC Genome Browser database: update 2011.. Nucleic Acids Res.

[53] Trapnell C, Pachter L, Salzberg SL (2009). TopHat: discovering splice junctions with RNA-Seq.. Bioinformatics.

[54] Langmead B, Trapnell C, Pop M, Salzberg SL (2009). Ultrafast and memory-efficient alignment of short DNA sequences to the human genome.. Genome Biol.

[55] Li H, Handsaker B, Wysoker A, Fennell T, Ruan J (2009). The Sequence Alignment/Map format and SAMtools.. Bioinformatics.

[56] Trapnell C, Williams BA, Pertea G, Mortazavi A, Kwan G (2010). Transcript assembly and quantification by RNA-Seq reveals unannotated transcripts and isoform switching during cell differentiation.. Nat Biotechnol.

[57] Bernatavichute YV, Zhang X, Cokus S, Pellegrini M, Jacobsen SE (2008). Genome-wide association of histone H3 lysine nine methylation with CHG DNA methylation in Arabidopsis thaliana.. PLoS ONE.

[58] Bulow L, Brill Y, Hehl R (2010). AthaMap-assisted transcription factor target gene identification in Arabidopsis thaliana.. Database (Oxford).

