# Supplementary material for: Dynamic Deposition of Histone Variant H3.3 Accompanies Developmental Remodeling of the Arabidopsis Transcriptome
Source: PLoS Genet. 2012 May 3;8(5):e1002658. doi: 10.1371/journal.pgen.1002658 (PMC3342937; doi:10.1371/journal.pgen.1002658)
Supplement: Table S1 — Overview of sequencing libraries analyzed. (DOC) [file pgen.1002658.s008.doc]

**Table S1. Overview of sequencing libraries analyzed.**

| **Name** | **Ab.** | **Strain** | **Condition** | **Rep.** | **PF reads** | **Mapped reads** | **Used in** |
| --- | --- | --- | --- | --- | --- | --- | --- |
| CAT005 | GFP | H3.3-GFP (HTR5) | Dividing Cells | 1 | 27,222,359 | 11,904,341 | FigS1 |
| CAT015 | GFP | H3.3-GFP (HTR5) | Dividing Cells | 2 | 26,845,209 | 20,638,245 | Fig1-2-3-S1-S3-S6-S7 |
| CAT009 | GFP | H3.1-GFP (HTR13) | Dividing Cells | 1 | 31,372,169 | 8,463,436 | FigS1 |
| CAT019 | GFP | H3.1-GFP (HTR13) | Dividing Cells | 2 | 23,197,312 | 16,148,763 | Fig1-2-3-S1-S3-S6-S7 |
| CAT008 | H3 | H3.1-GFP (HTR13) | Dividing Cells | 1 | 23,867,461 | 6,073,683 | FigS1 |
| CAT014 | H3 | H3.3-GFP (HTR5) | Dividing Cells | 2 | 27,985,047 | 17,018,135 | Fig1-2-3-S1-S3-S4-S6-S7 |
| CAT007 | IgG | H3.1-GFP (HTR13) | Dividing Cells | 1 | 25,503,399 | 492,277 | FigS1 |
| CAT013 | IgG | H3.3-GFP (HTR5) | Dividing Cells | 2 | 26,448,814 | 15,147,753 | Fig1-2-3-S1-S3-S4-S6-S7 |
| CAT006 | GFP | H3.3-GFP (HTR5) | Non-dividing Cells | 1 | 26,728,418 | 8,716,707 | FigS1 |
| CAT018 | GFP | H3.3-GFP (HTR5) | Non-dividing Cells | 2 | 27,008,441 | 20,715,803 | Fig3-S1-S2-S3-S5-S7 |
| CAT012 | GFP | H3.1-GFP (HTR13) | Non-dividing Cells | 1 | 28,578,344 | 10,504,903 | FigS1 |
| CAT020 | GFP | H3.1-GFP (HTR13) | Non-dividing Cells | 2 | 24,333,562 | 16,317,664 | Fig3-S1-S2-S3-S5-S7 |
| CAT011 | H3 | H3.1-GFP (HTR13) | Non-dividing Cells | 1 | 21,124,684 | 7,531,364 | FigS1 |
| CAT017 | H3 | H3.3-GFP (HTR5) | Non-dividing Cells | 2 | 27,591,959 | 18,027,433 | Fig3-S1-S2-S3-S5-S7 |
| CAT010 | IgG | H3.1-GFP (HTR13) | Non-dividing Cells | 1 | 27,248,726 | 228,484 | FigS1 |
| CAT016 | IgG | H3.3-GFP (HTR5) | Non-dividing Cells | 2 | 27,054,492 | 18,133,455 | Fig3-S1-S2-S3-S5-S7 |
| RAT001 | RNA-Seq | H3.3-GFP (HTR5) | Non-dividing Cells | 1 | --- | 34,870,976 | Fig2-3-S4-S5-S7 |
| RAT002 | RNA-Seq | H3.3-GFP (HTR5) | Dividing Cells | 1 | --- | 39,430,750 | Fig2-3-S4-S5-S7 |
| RAT003 | RNA-Seq | H3.1-GFP (HTR13) | Non-dividing Cells | 1 | --- | 34,586,466 | Fig2-3-S4-S5-S7 |
| RAT004 | RNA-Seq | H3.1-GFP (HTR13) | Dividing Cells | 1 | --- | 39,085,703 | Fig2-3-S4-S5-S7 |
