# Supplementary material for: Dynamic Deposition of Histone Variant H3.3 Accompanies Developmental Remodeling of the Arabidopsis Transcriptome
Source: PLoS Genet. 2012 May 3;8(5):e1002658. doi: 10.1371/journal.pgen.1002658 (PMC3342937; doi:10.1371/journal.pgen.1002658)
Supplement: Table S2 — Expression of the top-most cell-cycle dependent and independent genes in dividing and non-dividing tissue. CycleBase ranks were extracted from www.cyclebase.org with ranks from 1 to 20,945 reflecting the magnitude of cell-cycle dependent regulation. FPKM values are mean values obtained from the HTR5::GFP and HTR13::GFP mRNA-Seq libraries. (DOC) [file pgen.1002658.s009.doc]

**Table S2: Expression of the top-most cell-cycle dependent and independent genes in dividing and non-dividing tissue.**

| **Category** | **Gene** | **CycleBase Rank** | **Dividing (FPKM)** | **Non-dividing (FPKM)** | **Ratio Div/Non** |
| --- | --- | --- | --- | --- | --- |
| **Cell-cycle regulated** | AT1G18250 | 1 | 15.52 | 0.42 | 36.99 |
|  | AT3G54560 | 2 | 47.34 | 9.14 | 5.18 |
|  | AT4G21820 | 3 | 2.18 | 0.18 | 11.83 |
|  | AT5G23420 | 4 | 15.87 | 5.88 | 2.70 |
|  | AT4G28430 | 5 | 1.68 | 0.00 | high |
|  | AT3G12870 | 6 | 10.19 | 0.00 | high |
|  | AT4G23800 | 7 | 33.27 | 1.03 | 32.29 |
|  | AT1G02730 | 8 | 18.23 | 0.81 | 22.63 |
| **Cell-cycle regulated** | AT1G01910 | 20,938 | 31.96 | 23.07 | 1.39 |
|  | AT3G63270 | 20,939 | 11.41 | 5.59 | 2.04 |
|  | AT1G66670 | 20,940 | 44.78 | 51.24 | 0.87 |
|  | AT5G49230 | 20,941 | 15.93 | 11.83 | 1.35 |
|  | AT1G09790 | 20,942 | 0.00 | 0.00 | 1.00 |
|  | AT5G46750 | 20,943 | 28.63 | 22.74 | 1.26 |
|  | AT3G60600 | 20,944 | 169.42 | 101.19 | 1.67 |
|  | AT4G19540 | 20,945 | 7.44 | 6.78 | 1.10 |
